# Coil Probe Dimension and Uncertainties During Measurements of Nonuniform ELF Magnetic Fields

**DOI:** 10.6028/jres.098.024

**Published:** 1993

**Authors:** Martin Misakian

**Affiliations:** National Institute of Standards and Technology, Gaithersburg, MD 20899-0001

**Keywords:** appliance, coil probe, dipole field, magnetic field, measurement, measurement uncertainty, power frequency, residential, transportation systems, work place

## Abstract

Comparisons are made between the calculated average magnetic flux density for single-axis and three-axis circular coil probes and the calculated magnetic flux density at the center of the probes. The results, which are determined as suming a dipole magnetic field, provide information on the uncertainty associated with measurements of nonuniform extremely low frequency (ELF) magnetic fields produced by some electrical appliances and other electrical equipment.

## 1. Introduction

The concern in the mid 1970s regarding health effects from exposure to electric and magnetic fields in the vicinity of power lines has shifted in recent years to health effect concerns from exposure to power frequency magnetic fields in residences, the work place, and in transportation systems [1–3]. The magnetic fields in these environments can be highly nonuniform, particularly near electrical equipment such as motors, transformers and heating elements. This paper considers the difference between the calculated average magnetic flux density, *B*_av_, as determined using magnetic field meters with single-axis and three-axis circular coil probes, and the calculated magnetic flux density at the center of the probes, *B*_0_ assuming the field is produced by a small loop of alternating current, i.e., a magnetic dipole. The magnetic dipole field is chosen as the relevant field because to a good approximation its geometry simulates the field geometry of many electrical appliances and equipment [4]. The difference between *B*_av_ and *B*_0_ can be regarded as a source of measurement uncertainty because the center of the probe is normally considered the measurement location. While differences between *B*_av_ and *B*_0_ will be small in many situations, e.g., near ground level in the vicinity of power lines where the field changes slowly, the difference can become significant in the highly nonuniform magnetic fields close to electrical equipment.

In this paper, two comparisons are made: (1) the maximum average magnetic field determined using a single-axis probe, *B*_av1_, with *B*_0_ as a function of *r/a* where *r* is the distance between the magnetic dipole and the center of the probe, and *a* is the radius of the probe, and (2) the resultant magnetic field determined using a three-axis probe with *B*_0_ as a function of *r/a*. The resultant magnetic field, *B*_av3_, is defined as [5]

$$B_{\mathrm{av}3}=\sqrt{B_{1}^{2}B_{2}^{2}B_{3}^{2}},$$ (1)

where *B*_1_, *B*_2_, and *B*_3_ are average magnetic field components as measured by three orthogonally oriented coil probes.

Comparison (1) is made because maximum magnetic field values are sometimes measured, using single-axis field meters, to characterize the magnetic field [5,6]. However, for a given value of *r/a*, it will be seen that the difference between *B*_av1_ and *B*_0_ will be a function of the orientation of the magnetic dipole relative to the probe. Because the relative orientation is not known during most measurements, what is of interest is the largest difference between *B*_av1_ and *B*_0_ for a given value of *r/a.* This largest difference will be designated Δ*B*_max1_.

**Fig. 1:**
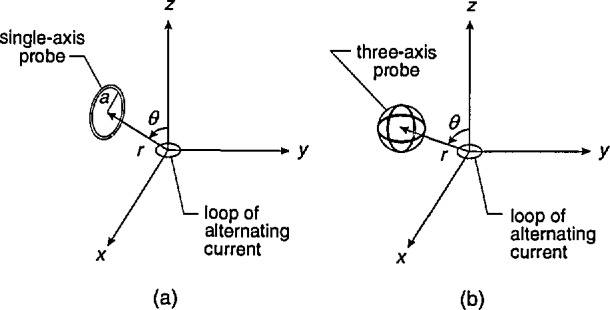
(a) Single-axis and (b) three-axis circular coil probes in dipole magnetic field produced by small loop of current.

The quantity Δ*B*_max1_ is determined in the following way. The single-axis probe is rotated for fixed values of *r/a* and the spherical coordinate, *θ*, [Fig. 1(a)] until the largest average magnetic field, *B*_av1_, is found. This value of *B*_av1_ is compared with the magnetic field at the center of the probe, *B*_0_ and the difference is recorded. The orientation of the magnetic dipole with respect to the probe is then varied by moving the probe to another location while keeping *r/a* fixed, i.e., by changing *θ* in Fig. 1(a). The probe is rotated again until the largest average magnetic field, *B*_av1_, is found. *B*_av1_ is again compared with the magnetic field at the center of the probe, *B*_0_, and the difference is recorded. This process is repeated for other dipole orientations (i.e., angle *θ*) until the largest difference, Δ*B*_max1_, is found. An example of this process is shown in Sec. 3.1.

Comparison (2) is made as a three-axis probe is rotated about three axes parallel to the three Cartesian coordinates *x, y*, and *z*. The difference between *B*_av3_ and *B*_0_ will vary as a function of rotation angle, but what will be of interest again is the largest difference, Δ*B*_max3_, for a given *r/a.* Also as for comparison (1), because the relative orientations of the magnetic dipole and the three-axis probe will be unknown in most measurement situations, *B*_av3_ will be examined as a function of *r/a* and the spherical coordinate, *θ*, in order to determine the largest difference, Δ*B*_max3_.

## 2. Expressions for Average Magnetic Flux Density

In the derivations given below, it is assumed that the cross sectional area of the wire in the coil probes and the opposing magnetic field produced by current induced in the probes are negligible. In addition, we assume for the three-axis probe that the three orthogonally oriented coils have circular cross sections of equal area. These assumptions either can be met in practice or can be taken into account via a calibration process.

### 2.1 Single-Axis Probe

The average magnetic flux density, *B*_av_, for a single coil probe with cross sectional area *A* is given by

$$B_{\text{av}}=\frac{1}{A}\iint_{A}B\cdot \hat{n}dA,$$ (2)

where d*A* is an element of probe area, $\hat{n}$ is a unit vector perpendicular to *A*, and ***B*** is the magnetic flux density. In spherical coordinates, the magnetic flux density for a small current loop of radius *b* is [7]

$$B=\frac{\mu_{0}Ib^{2}}{2r^{3}}\cos\theta {\hat{u}}_{r}+\frac{\mu_{0}Ib^{2}}{4r^{3}}\sin\theta {\hat{u}}_{\theta },$$ (3)

where *μ*_0_ is the permeability of vacuum, *I* is the alternating current, and *û_r_* and *û_θ_* are unit vectors in the directions of increasing *r* and *θ*, respectively. The assumption is made that *b < <r* and the sinusoidal time dependence has been suppressed. The magnitude of the vector ***B*** given by Eq. (3) is *B*_0_.

For our purposes, it is convenient to express ***B*** in terms of Cartesian coordinates. This is accomplished by using the following relations between spherical and Cartesian unit vectors and coordinates [8] in Eq. (3):

$$\begin{array}{l} {\hat{u}}_{r}=\hat{i}\sin\theta \cos\phi +\hat{j}\sin\theta \sin\phi +\hat{k}\cos\theta \\ {\hat{u}}_{B}=\hat{i}\cos\theta \cos\phi +\hat{j}\cos\theta \sin\phi -\hat{k}\sin\theta \\ x=r\sin\theta \cos\phi \\ y=r\sin\theta \sin\phi \\ z=r\cos\theta . \end{array}$$ (4)

After some algebra, ***B*** can be expressed as

$$B=\hat{i}\frac{3Cxz}{2r^{5}}+\hat{j}\frac{3Cyz}{2r^{5}}+\hat{k}\frac{C}{2r^{3}}\left(\frac{3z^{2}}{r^{2}}-1\right),$$ (5)

where $r=\sqrt{x^{2}+y^{2}+z^{2}}$ and *C* is the constant *μ*_0_*Ib*^2^/2.

**Fig. 2:**
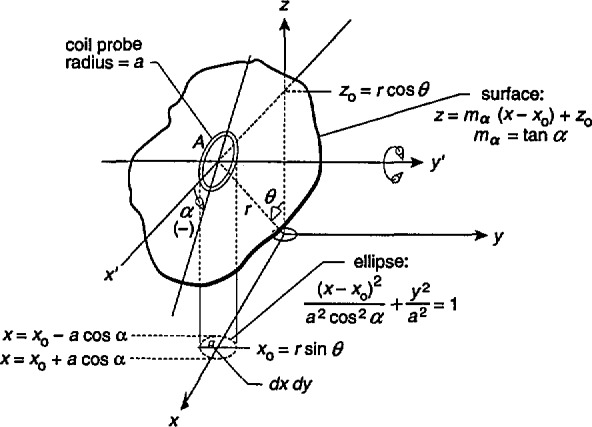
Circular coil probe shown as part of a surface described by the equation *z=m_α_*(*x−x*_0_*)+z*_0_. The rotation of the probe corresponds to changing the slope of the surface, *m_α_.* The projection of the probe cross sectional area onto the *x-y* plane will be an ellipse for *α*≠0. The range of *α* is −90°<*α*< 90°.

To obtain an expression for *B*_av_, we consider without loss of generality a probe with its center at *x=x*_0_,*y*=0, and *z=z*_0_ as shown in Fig. 2. We restrict the orientation of the probe so that its area is bisected by the *x-z* plane and first consider rotations of the probe about an axis parallel to the *y*-axis, i.e., the *y*′-axis shown in Fig. 2. For these conditions, the area of the coil probe, *A*, will be part of the surface given by the equation

$$z=m_{\alpha }\left(x-x_{0}\right)+z_{0},$$ (6)

where *α* is the angle of rotation, *m_α_* = tan *α*, *x*_0_=*r* sin *θ*, and *z_0_=r* cos *θ.* The rotation of the probe corresponds to the rotation of this surface about the *y*′-axis, i.e., changing the slope of the surface (*m_α_*) described by Eq. (6). It should be noted that the angle of rotation, *α*, shown in Fig. 2 is in the negative direction.

The unit vector perpendicular to the probe surface, $\hat{n}$, is found by first taking the gradient [9] of the surface given by Eq. (6), *∇F*(*x,z*), where *F*(*x,z*)*=z−m_α_*(*x−x*_0_)*−z*_0_ and normalizing it to unit value. This leads to

$$\hat{n}=\left(-m_{\alpha }\hat{i}+\hat{k}\right)/\sqrt{m_{\alpha }^{2}+1}.$$ (7)

The element of area, d*A*, is [10]

$$dA=\sqrt{{\left(\frac{\delta z}{\delta x}\right)}^{2}+{\left(\frac{\delta z}{\delta y}\right)}^{2}+1}dxdy=\sqrt{m_{\alpha }^{2}+1}dxdy.$$ (8)

where d*x* d*y* is an element of area in the *x-y* plane bounded by the projection of the probe cross section onto the *x-y* plane (Fig. 2).

Combining Eqs. (2), (5), (7), and (8), the expression for *B*_av_ becomes

$$B_{\text{av}}=\frac{C}{2\pi a^{2}}\int_{x}\int_{y}\left\{\frac{-3xzm_{\alpha }}{r^{5}}+\frac{1}{r^{3}}\left(\frac{3z^{2}}{r^{2}}-1\right)\right\}dxdy.$$ (9)

By substituting for *z* in Eq. (9) using Eq. (6), the integrand becomes a function of *x* and *y.* The integration is first carried out analytically [11] over the variable *y* with (from Fig. 2)

$$\begin{array}{l} -\sqrt{a^{2}-{\left(\left(x-x_{0}\right)/\cos\;\alpha \right)}^{2}}\leq y\leq \\ \sqrt{a^{2}-{\left(\left(x-x_{0}\right)/\cos\;\alpha \right)}^{2}}. \end{array}$$

The resulting expression for *B*_av_ is

$$\begin{array}{l} B_{\text{av}}=-\frac{C}{\pi a^{2}}\int dx\left(m_{\alpha }xP+P^{2}\right)\\ \{\frac{\sqrt{a^{2}-{\left(\frac{x-x_{0}}{\cos\;\alpha }\right)}^{2}}}{\left(x^{2}+Q^{2}\right){\left(x^{2}+Q^{2}+a^{2}-{\left(\frac{x-x_{0}}{\cos\;\alpha }\right)}^{2}\right)}^{3/2}}+\\ \frac{2\sqrt{a^{2}-{\left(\frac{x-x_{0}}{\cos\;\alpha }\right)}^{2}}}{{\left(x^{2}+Q^{2}\right)}^{2}{\left(x^{2}+Q^{2}+a^{2}-{\left(\frac{x-x_{0}}{\cos\;\alpha }\right)}^{2}\right)}^{1/2}}\}-\\ \frac{C}{\pi a^{2}}\int dx\frac{\sqrt{a^{2}-{\left(\frac{x-x_{0}}{\cos\;\alpha }\right)}^{2}}}{\left(x^{2}+Q^{2}\right){\left(x^{2}+Q^{2}+a^{2}-{\left(\frac{x-x_{0}}{\cos\;\alpha }\right)}^{2}\right)}^{1/2}}, \end{array}$$ (10)

where *P* = (*z*_0_−*m_α_x*_0_) and *Q*^2^=(*m_α_x + P*)^2^.

The integration over *x* is then performed numerically using Simpson’s Rule with the limits of integration given by (Fig. 2)

$$x_{0}-a\cos\;\alpha \leq x\leq x_{0}+a\cos\;\alpha ,$$

where *α* is restricted to −90° < *α* < 90°.

*B*_av_ is evaluated for fixed values of *θ* and *r/a* as *α* is varied until a maximum average flux density, *B*_av1_, is found. *B*_av1_ is then compared with *B*_0_. As noted earlier, the process is repeated for the same *r/a* but different values of *θ* until the largest difference, *ΔB*_max1_, is determined. Because we are seeking the maximum value of *B*_av_, we do not consider further rotations of the probe because once *B*_av1_ is found, additional rotations are expected to lead to smaller values of *B*_av_. This is most readily seen at *θ* equal to 0° and 90° for all values of *r/a*. *B*_av1_ occurs at *α* = 0° and rotating the probe further results in smaller values of *B*_av_.

### 2.2 Three-Axis Probe

In this section, expressions are developed for the average magnetic flux density for each coil of a three-axis probe as the probe is rotated about axes which are parallel to the *x*-, *y*-, and *z*-axes. Afterwards, for fixed values of *θ* and *r/a*, the average magnetic field values from the three orthogonally oriented probes are combined according to Eq. (1) to obtain *B*_av1_ which is then compared with *B*_0_. As before, the process is repeated for different values of *θ* until the maximum difference between *B*_av3_ and *B*_0_, *ΔB*_max3_, is found.

It is noted that combinations of rotations about the different axes will not be possible using the expressions that are developed. That is, it will not be possible to calculate *B*_av3_ following rotations about two or three axes. This represents a limitation on the results and prevents us from learning whether there are significant effects on the value of *ΔB*_max3_ due to multiple rotations. Nevertheless, the departures from *B*_0_ that are determined from rotations about each of the three axes will let us know what differences are possible as a function of *r/a.*

**Fig. 3:**
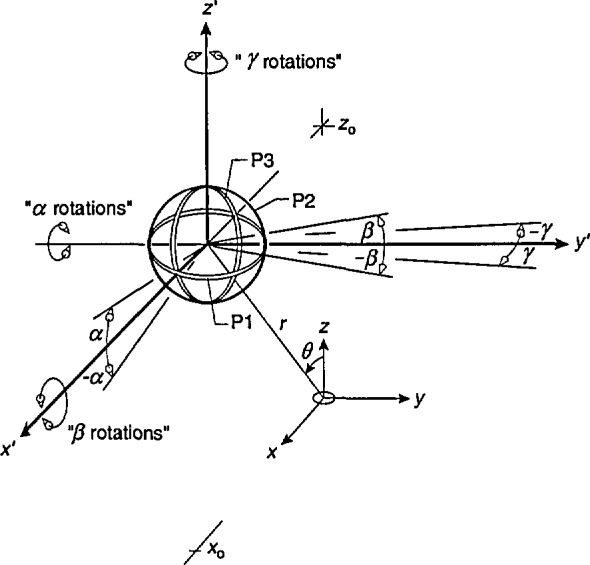
Geometry for three-axis probe with center of probe in *x-z* plane. Varying the angle *α* result in rotations of probes P1 and P2 about the *y*′-axis (“*α* rotations”) while orientation of probe P3 with respect to the dipole remains unchanged. Varying the angle *β* results in rotations of probes P1 and P3 about the *x*′-axis (“*β* rotations”) while orientation of probe P2 remains unchanged. Varying the angle *γ* results in rotations of probes P2 and P3 about the *z*′-axis (“*γ* rotations”) while orientation of probe P1 remains unchanged.

Figure 3 shows the orientation of the probe with respect to the magnetic dipole before rotations about each axis are considered. As in the previous section, the center of the probe is located at *x =x*_0_, *y*=0, and *z =z*_0_. The individual probes are labelled P1, P2, and P3, and initially P1 lies in the *x′-y′* plane, P2 is in the*y′*-*z′* plane, and P3 is in the *x′*-*z'* plane. When rotations are performed about the *x'*-axis (rotation angle *β*), the angle that P2 makes with the magnetic field remains unchanged. Similarly, rotations about the *y'*-axis (rotation angle *α*) and *z'*-axis (rotation angle *γ*) leave P3 and P1, respectively, “unchanged.” This means that for constant values of *r/a* and *θ*, the average flux density values for these “fixed” probes remain constant as rotations of the probe occur.

We begin the derivation for the three-axis probe by noting that part of the problem has already been solved in the previous section. That is, the expression for *B*_av_ following rotations about the *y*′-axis (*α* rotations) is given by Eq. (10). This expression is used to calculate the average flux density for probes P1 and P2 by considering pairs of the angle *α* which differ by 90°. The average flux density for the third probe, P3, is zero for this case because no component of the magnetic field is perpendicular to the area of the probe for any value of *α* or *θ.*

**Fig. 4:**
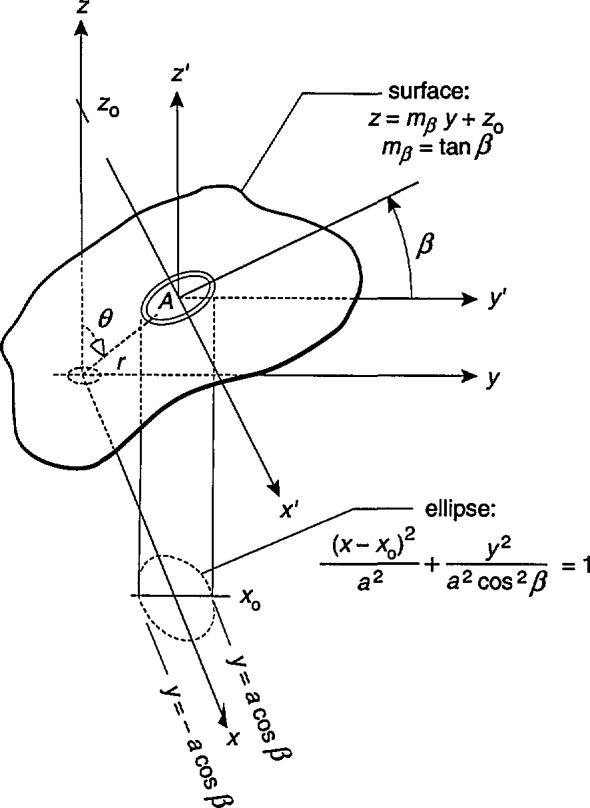
Circular coil probe shown as part of a surface described by the equation *z=m_β_y+z*_0_. The rotation of the probe corresponds to changing the slope of the surface, *m_β_.* The projection of the probe cross sectional area onto the *x-y* plane will be an ellipse for *β*≠0. The range of *β* is −90° < *β* < 90°.

The derivations for *B*_av_ following *β* or *γ* rotations parallel the derivation for the *α* rotations. Examining the case of *β* rotations first, and referring to Fig. 4, it can be seen that the probe area, *A*, is part of the surface given by the equation

$$z=m_{\beta }y,+z_{0},$$ (11)

where m*_β_* = tan *β* and *z*_0_=*r* cos *θ.* The rotation of the probe corresponds to rotation of this surface about the *x*′-axis or alternatively, changing its slope, m*_β_.* The unit vector normal to this surface is

$$\hat{n}=\left(-m_{\beta }\hat{j}+\hat{k}\right)/\sqrt{m_{\beta }^{2}+1},$$ (12)

and the element of area, d*A*, is

$$dA=\sqrt{{\left(\frac{\delta z}{\delta x}\right)}^{2}+{\left(\frac{\delta z}{\delta y}\right)}^{2}+1}dxdy=\sqrt{m_{\beta }^{2}+1}dxdy.$$ (13)

Combining Eqs. (2), (5), (12), and (13), the expression for the average flux density following *β* rotations is

$$\begin{array}{l} B_{\text{av}\beta }=\frac{1}{\pi a^{2}}\int_{y}\int_{x}B\cdot \hat{n}dA=\\ -\frac{3Cm_{\beta }}{2\pi a^{2}}\int_{y}\int_{x}\frac{yz}{r^{5}}dxdy+\\ \frac{C}{2\pi a^{2}}\int_{y}\int_{x}\left(\frac{3z^{2}}{r^{5}}-\frac{1}{r^{3}}\right)dxdy. \end{array}$$ (14)

Substituting for *z* in Eq. (14) using Eq. (11), the integrands become, recalling that $r=\sqrt{x^{2}+y^{2}+z^{2}}$, a function of *x* and *y*. The integration is first carried out analytically over the variable *x* with (Fig. 4)

$$x_{0}-\sqrt{a^{2}-{\left(y/\cos\beta \right)}^{2}}\leq x\leq x_{0}+\sqrt{a^{2}-{\left(y/\cos\beta \right)}^{2}}.$$

The *y*-integration is performed numerically with the limits of integration given by (Fig. 4)

−acosβ≤y≤acosβ,

where *β* is restricted to −90° < *β* < 90°.

Equation (14) is used to calculate the average flux density for probes P1 and P3 by considering pairs of the angle *β* which differ by 90°. The average flux density from the remaining probe, P2, remains constant during the *β* rotations and is determined from the expression for average flux density following *γ* rotation (*γ*=0)—which is developed below. The reader is cautioned that during the numerical integration over the variable *y*, the denominator in the integrand vanishes for *y* = 0 when *θ* = 90°.

**Fig. 5:**
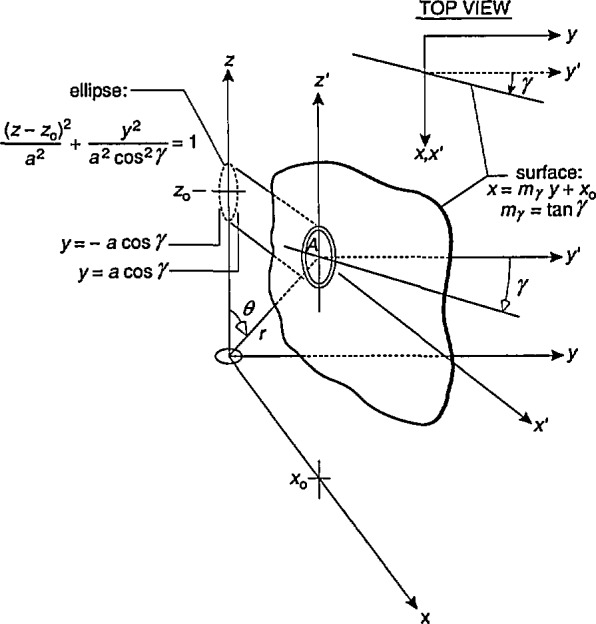
Circular coil probe shown as part of a surface described by the equation *x =m_γ_y + x*_0_. The rotation of the probe corresponds to changing the slope of the surface, *m_γ_.* The projection of the probe cross sectional area onto the *y-z* plane will be an ellipse for *γ*≠0. The range of *γ* is −90° <*γ* < 90°.

In deriving the expression for average flux density following *γ* rotations, we note as shown in Fig. 5 that the probe area, *A*, is part of the surface given by the equation

$$x=m_{\gamma }y+x_{0},$$ (15)

where *m_γ_* = tan*γ* and *x*_0_*=r* sin *θ*. The rotation of the probe corresponds to rotation of this surface about the *z*′-axis, i.e., changing the slope of the surface, *m_γ_.* The unit vector normal to this surface is

$$\hat{n}=\left(\hat{i}-m_{\gamma }\hat{j}\right)/\sqrt{m_{\gamma }^{2}+1},$$ (16)

and the element of area, d*A*, is

$$dA=\sqrt{{\left(\frac{\delta x}{\delta y}\right)}^{2}+{\left(\frac{\delta x}{\delta z}\right)}^{2}+1}dydz=\sqrt{m_{\gamma }^{2}+1}dydz.$$ (17)

It should be noted that the projection of the probe’s cross sectional area, unlike the previous two cases, is onto the *y*-*z* plane for *y* rotations (the projections for *α* and *β* rotations were onto the *x-y* plane) and this fact affects the partial derivatives in the expression for d*A*, Eq. (17).

From Eqs. (2), (5), (16), and (17), the expression for average flux density following *γ* rotations is

$$\begin{array}{l} B_{\text{av}\gamma }=\frac{1}{\pi a^{2}}\int_{y}\int_{z}B\cdot \hat{n}dA=\\ \frac{3C}{2\pi a^{2}}\int_{y}\int_{z}\left(\frac{xz}{r^{5}}-\frac{m_{\gamma }yz}{r^{5}}\right)dzdy. \end{array}$$ (18)

By substituting for *x* in Eq. (18) using Eq. (15), the integrand becomes a function of *y* and *z*. The integration is first carried out analytically over the variable *z* with (Fig. 5)

$$z_{0}-\sqrt{a^{2}-{\left(y/\cos\gamma \right)}^{2}}\leq z\leq z_{0}+\sqrt{a^{2}-{\left(y/\cos\gamma \right)}^{2}}.$$

The *y*-integration is performed numerically with the limits of integration given by

−acosγ≤y≤acosγ,

where *γ* is restricted to −90° < *γ* < 90°.

Equation (18) is used to calculate the average flux density for probes P2 and P3 by considering pairs of the angle, *γ*, which differ by 90°. The average flux density from the remaining probe, P1, remains constant during the *γ* rotations and is calculated from the expression for average flux density for *α* rotations [Eq. (10)] with *α* set equal to zero.

## 3. Results of Calculations

### 3.1 *ΔB*_max1_ for Single-Axis Probe

**Fig. 6:**
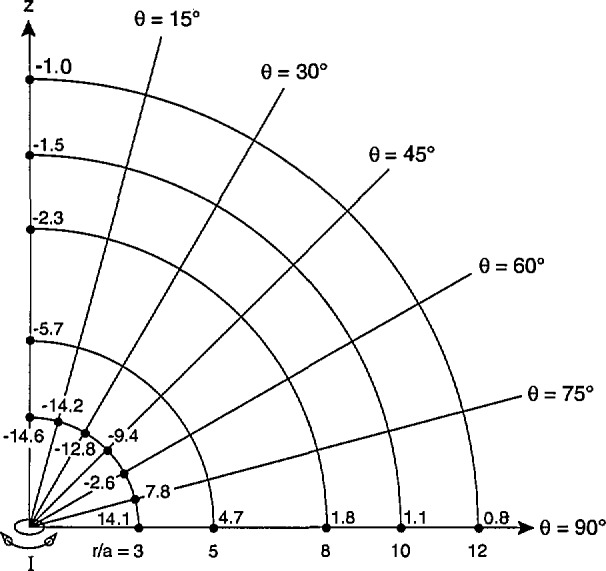
Differences between values of *B*_avi_ and B_0_, in percent, for different locations of single-axis probe relative to magnetic dipole which is aligned along the *z*-axis. For a given value of *r/a*, where *r/a* is ⩾ 3, the largest difference, *ΔB*_max1_, is negative and occurs when the probe is located along the *z*-axis.

Using Eq. (10) and following the procedure described after Eq. (1), values of the maximum average magnetic field, *B*_av1_ for fixed values of *r/a* and *θ* were calculated and compared with the corresponding value of *B*_0_. Figure 6 shows the differences in percent between *B*_av1_ and *B*_0_ for *r/a* = 3 and for representative values of *θ* between 0° and 90° (because of symmetry arguments, one can infer the corresponding percentages for *θ* between 90° and 180°). The largest difference, *ΔB*_max1_, is −14.6% and occurs when the single-axis probe is located along the axis of the magnetic dipole, i.e., the *z*-axis. The negative difference between *B*_av1_ and *B*_0_ decreases as *θ* increases and turns positive near *θ* = 90°. This pattern also occurs for other values of *r/a* greater than 3. Figure 6 also shows the largest negative and positive differences in percent for *r/a* equal to 5, 8, 10, and 12. The largest negative differences must be considered part of the measurement uncertainty when the probe-dipole geometry is unknown, which will be the case for example when magnetic field measurements are performed near many appliances. A tabulation of *ΔB*_max1_, as a function of r/a is given in Table 1.

The calculations are not carried out for large values of *r/a* because the accuracy requirements for magnetic field measurements near appliances and other electrical equipment either have not been set or are not great. For example, the uncertainty tentatively allowed during calibration of magnetic field meters used for measuring magnetic fields near visual display terminals is ± 5% [12].

### 3.2 *ΔB*_max3_ for Three-Axis Probe

The differences between *B*_av3_ and *B*_0_ are considered in three steps. First, values of *B*_av3_ are calculated following *α* rotations using Eq. (10) and compared with *B_0_* for fixed values of *r/a* and representative values of *θ* between 0° and 90°. As noted earlier, the largest difference between *B*_av3_ and *B*_0_ at each point, a “local maximum difference,” is recorded. In the discussion that follows, the “local maximum difference,” will be referred to simply as the “difference.” The above procedure is repeated for *β* and *γ* rotations.

**Fig. 7:**
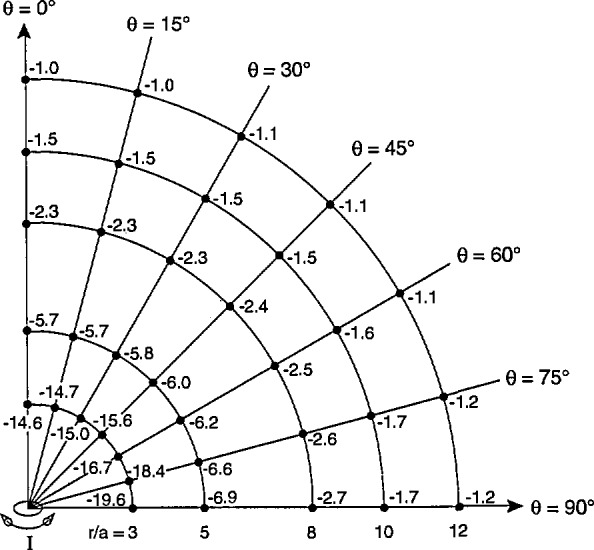
Differences between values of *B*_av3_ and *B*_0_ in percent, for different locations of three-axis probe relative to magnetic dipole, following *α* rotations. The differences are always negative and the greatest difference following *α* rotations occurs for *θ* = 90°.

The differences between *β*_av3_ and *β*_0_ following *α* rotations are plotted in Fig. 7 for *r/a* equal to 3, 5, 8, 10, and 12. The numbers in Fig. 7 represent differences in percent. The pattern observed for all values of *r/a* is that the difference at a point following *α* rotation is always negative and becomes more negative as *θ* increases to 90°.

**Fig. 8:**
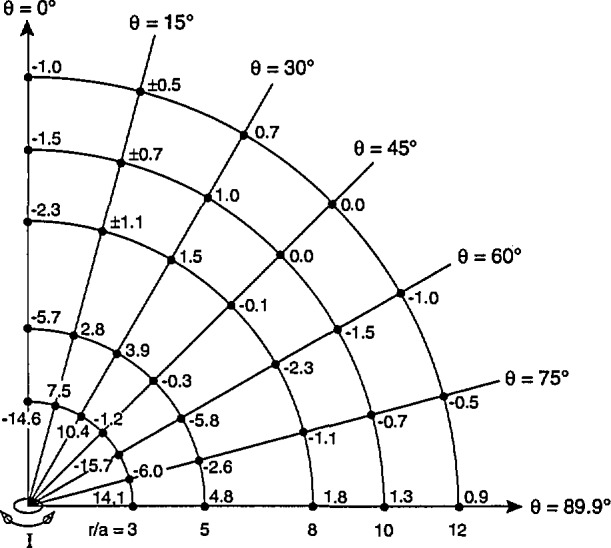
Differences between values of *B*_av3_ and *B*_0_ in percent, for different locations of three-axis probe relative to magnetic dipole, following *β* rotations. The differences vary in sign depending on angle *θ.* For *θ* = 15°, there are several cases (indicated with the ± sign) for which the largest negative and positive differences are equal in magnitude.

When the difference calculations are performed for *β* rotations using Eq. (14) and Eq. (18) (*γ* set equal to zero), a different pattern emerges. The differences between *B*_av3_ and *B*_0_ are observed to change in sign at different points as shown in Fig. 8. By comparing the results in Figs. 7 and 8, it can be seen that, except for *θ* = 0, the differences following *β* rotations are all less than the corresponding (i.e., same *r/a* and *θ* values) differences following *α* rotations. Although not indicated in Fig. 8 calculations show that for a given value of *r/a* ≥3, the differences between *B*_av3_ and *B*_0_ following *β* rotations for all values of *θ* are less than the difference following *α* rotations when *θ* = 90°.

**Fig. 9:**
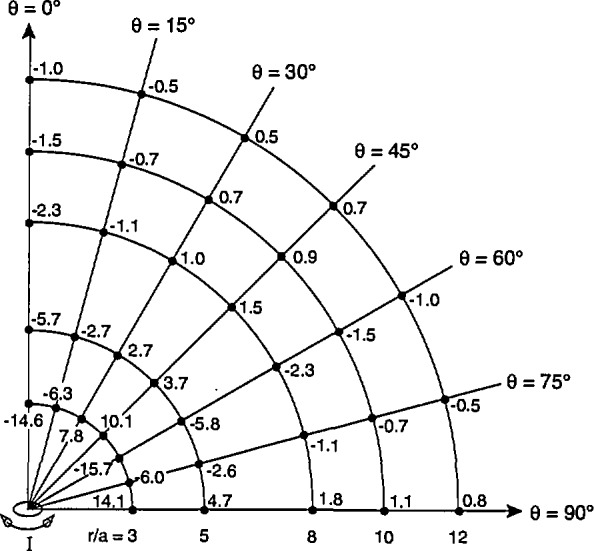
Differences between values of *B*_av3_ and B_0_ in percent, for different locations of three-axis probe relative to magnetic dipole, following *γ* rotations. The differences vary in sign depending on angle *θ.*

Similar results occur following *γ* rotations when *B*_av3_ is calculated using Eq. (18) and Eq. (10) (*α* set equal to zero) and is compared with *B*_0_. Once again the differences between *B*_av3_ and *B*_0_ change in sign as shown in Fig. 9. Also, by comparing results in Figs. 7 and 9, it is seen that, except for *θ* = 0°, the differences following *γ* rotations are all less than the corresponding differences following *α* rotations. As for the case of *β* rotations, calculations show that for a given value of *r/a* ≥ 3, the largest difference following *γ* rotations will always be less than the difference following *α* rotations when *θ* = 90°. Therefore, of the three types of three-axis probe rotations considered, the greatest difference between *B*_av3_ and *B*_0_ can be found following *α* rotations and *ΔB*_max3_ occurs when *θ*=90° for a given value of *r/a.* Table 1 provides a listing of *ΔB*_max3_ values as a function of *r/a.*

## 4. Discussion of Results

Once it has been decided what constitutes an acceptable level of uncertainty during magnetic field measurements near electrical equipment, the information in Table 1 should be considered when taking into account the various sources of measurement uncertainty. For example, if maximum magnetic fields at a distance *r* from appliances are to be measured with a total uncertainty of less than ± 10%, magnetic field meters with probes having radii *a* such that *r/a ≈* 3 would immediately be considered unsuitable. Field meters with single-axis probes having radii such that *r/a* = 5 would be suitable if all other sources of uncertainty (e.g., calibration process, frequency response) amounted to about 8% or less, i.e., $\sqrt{5.7^{2}+8^{2}}=9.8$, where 5.7 is taken from Table 1 for *r/a* = 5.

The measurement uncertainties associated with using three-axis probes are less clear because we have considered only separate rotations about three axes to obtain the values of *ΔB*_max3_. The percentage differences in Table 1 indicate what uncertainties can occur but they may not be the largest uncertainties due to the averaging effects of the probe. However, until calculations can be devised which consider more complex rotations of three-axis probes, the *ΔB*_max3_ values in Table 1 can serve as a rough guide when deciding what are acceptable probe dimensions.

## 5. Acknowledgments

## Figures and Tables

**Table 1:** Values of *ΔB*_max1_ (single-axis probe) and *ΔB*_max3_ (three-axis probe) as a function of normalized distance (*r/a*) from magnetic dipole

*r/a*	*ΔB*_max1_ (%)	*ΔB*_max3_ (%)
3	−14.6	−19.6
4	−8.7	−10.8
5	−5.7	−6.9
6	−4.0	−4.8
7	−3.0	−3.5
8	−2.3	−2.7
9	−1.8	−2.1
10	−1.5	−1.7
11	−1.2	−1.4
12	−1.0	−1.2
13	−0.9	−1.0
14	−0.8	−0.9
15	−0.7	−0.8
